# Attracting Users to Online Health Communities: Analysis of LungCancer.net’s Facebook Advertisement Campaign Data

**DOI:** 10.2196/14421

**Published:** 2019-11-04

**Authors:** Lindsey N Horrell, Allison J Lazard, Amrita Bhowmick, Sara Hayes, Susan Mees, Carmina G Valle

**Affiliations:** 1 Department of Health Behavior Gillings School of Global Public Health University of North Carolina at Chapel Hill Chapel Hill, NC United States; 2 School of Media and Journalism University of North Carolina at Chapel Hill Chapel Hill, NC United States; 3 Lineberger Comprehensive Cancer Center University of North Carolina at Chapel Hill Chapel Hill, NC United States; 4 Health Union, LLC Philadelphia, PA United States; 5 Department of Nutrition Gillings School of Global Public Health University of North Carolina at Chapel Hill Chapel Hill, NC United States

**Keywords:** internet, health communication, social media, health promotion, health education

## Abstract

**Background:**

With growing numbers of adults turning to the internet to get answers for health-related questions, online communities provide platforms with participatory networks to deliver health information and social support. However, to optimize the benefits of these online communities, these platforms must market effectively to attract new members and promote community growth.

**Objective:**

The aim of this study was to assess the engagement results of Facebook advertisements designed to increase membership in the LungCancer.net online community.

**Methods:**

In the fall of 2017, a series of 5 weeklong Facebook advertisement campaigns were launched targeting adults over the age of 18 years with an interest in lung cancer to increase opt ins to the LungCancer.net community (ie, the number of people who provided their email to join the site).

**Results:**

The advertisements released during this campaign had a sum reach of 91,835 people, and 863 new members opted into the LungCancer.net community by providing their email address. Females aged 55 to 64 years were the largest population reached by the campaign (31,401/91,835; 34.29%), whereas females aged 65 and older were the largest population who opted into the LungCancer.net community (307/863; 35.57%). A total of US $1742 was invested in the Facebook campaigns, and 863 people opted into LungCancer.net, resulting in a cost of US $2.02 per new member.

**Conclusions:**

This research demonstrates the feasibility of using Facebook advertising to promote and grow online health communities. More research is needed to compare the effectiveness of various advertising approaches. Public health professionals should consider Facebook campaigns to effectively connect intended audiences to health information and support.

## Introduction

### Online Community Growth

Currently, 72% of adults seek health information on the Web, and 16% search for peers with similar health concerns [1]. Online communities can effectively extend health education [2,3] and facilitate social support [3,4] and have been linked to improved self-management [2] and enhanced health outcomes [3]. The number of online communities has grown substantially over the past decade, with countless websites increasing traffic from patients and caregivers through user-engaged communities [5]. Patients are motivated to join these communities to access support, advice, and accountability in reaching health goals [5-7]. Online community growth is crucial to meeting these user needs, as it builds communities’ pooled knowledge and increases access to quality informational and social support [5,8-11]. Larger online networks have the power of network effects—where more users increase the usefulness of the community [9]*.* For those seeking others with shared experiences, larger communities offer a greater number of individuals with the potential for cognitive empathy, particularly from people outside ones’ close network where sharing may cause emotional burden [12]*.* For staff overseeing these sites, limited evidence is available to guide community growth, which is known to be a time- and resource-intensive task [8].

### LungCancer.net

In this study, we reported the feasibility and cost-effectiveness of Facebook advertising to promote online community growth in the context of the LungCancer.net community. LungCancer.net provides patients and caregivers a platform to learn, educate, and connect with peers and health care professionals. The content published by LungCancer.net is written by patients, caregivers, and health professionals and supplemented by editorial content. In August 2017, LungCancer.net catered to 1575 users and sought to expand their community base through a series of social media advertisements. With 69% of US adults on Facebook and 74% of users on the site daily [13,14], Facebook seemed to be an ideal platform to promote community growth. The goal of this study was to assess the engagement results of Facebook advertisements designed to increase the number of opt ins to the LungCancer.net online community (ie, the number of users that provided their email to join the community).

## Methods

### Facebook Advertisement Campaign

**Figure 1:**
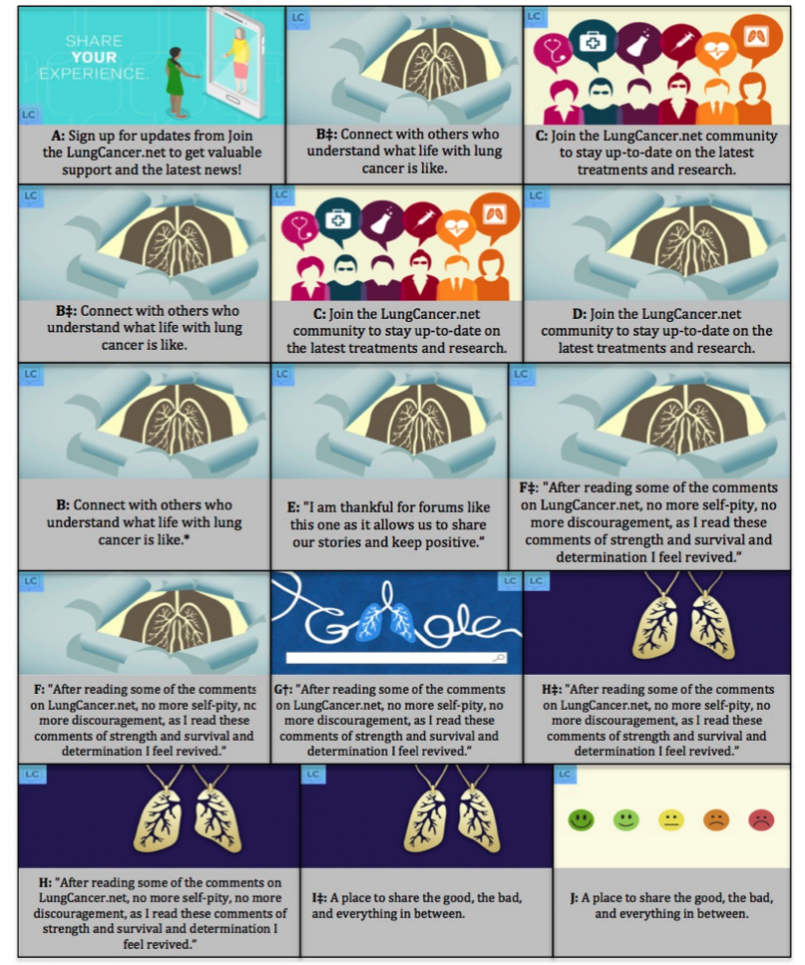
Facebook advertisement campaign images and text.

From August to December 2017, 5 weeklong Facebook campaigns were launched with the objective of increasing opt ins to LungCancer.net. Each campaign consisted of 3 unique advertisements that contained an image, a text, and a call to action (Figure 1). The visuals included 6 static images and 1 image in the Graphics Interchange Format (signaled with the “†” symbol in Figure 1). The text included messages crafted by community managers and quotes from members. The target audience was adults (18 years or older) with an interest in lung cancer–related content and/or Facebook pages. No other demographic variables were used to define the audience within the Facebook Ads Manager system. The budget for each advertisement was US $25 per day. Facebook utilizes a bidding cost system, and actual expenditures for each test averaged within 4% of the desired budget, with the exception of 1 outlying test, which was 19% below the budget.

### Advertisement Performance Measures

The performance of each advertisement was evaluated using metrics rooted in advertisement engagement frameworks [15-17]. According to McGuire’s Model of Persuasion, eliciting action begins with advertisement exposure and moves across a continuum of cognitive and behavioral responses [17]. Exposure in this campaign is operationalized as *impressions* (number of times the advertisement appears in News Feeds) and *reach* (number of individuals exposed to the advertisement). Frameworks proposed by Neiger et al [15] and Platt et al [16] were used to define low-to-high behavioral responses. As the goal of this campaign was to increase opt ins to the LungCancer.net community, *low user engagement* was defined as interacting with the advertisement through clicks (ie, reacting to the post, clicking a post link, or liking the LungCancer.net Facebook page), *medium user engagement* was defined as sharing or commenting on the advertisement, and *high user engagement* was defined as *opting in* or signing up for the LungCancer.net community. After each campaign, metrics (Table 1) were pulled for each advertisement, and advertisements with the lowest opt in cost were run with new advertisements during the next weeklong campaign. Advertisements with the lowest opt in cost during each weeklong campaign are signaled with the “‡” symbol in Figure 1.

**Table 1:** Facebook advertisement performance measures.

Level of performance	Level of engagement	Performance measure	Definition
Exposure	—^a^	Impressions	Number of times the advertisement appeared in News Feeds
Exposure	—	Reach	Number of individuals exposed to the Facebook advertisement
Engagement	Low	Reactions	Number of times people responded to an advertisement by clicking “like,” “love,” “wow,” “haha,” “sad,” or “angry”
Engagement	Low	Link clicks	Number of people who clicked a link on the Facebook advertisement
Engagement	Low	Page likes	Number of people who liked the LungCancer.net Facebook page
Engagement	Medium	Shares	Number of times people shared the advertisement
Engagement	Medium	Comments	Number of times people commented on the Facebook advertisement
Engagement	High	Opt ins	Number of people who signed up to join the LungCancer.net community

^a^Not applicable.

## Results

### Audience Demographics

Over the course of the 5 campaigns, the sum reach was 91,835 people, and 863 members opted in to the LungCancer.net community (ie, demonstrated high engagement; Table 2). Females between 55 and 64 years represented the largest population reached by the campaign (31,401/91,835; 34.29%), whereas females aged 65 years and older represented the largest population that opted in to the LungCancer.net community (307/863; 35.57%). Given that US $1742 was invested across the 5 campaigns, approximately US $2.02 was spent per opt in, and just over 1 cent was spent per exposure to the campaign.

### Advertisement Engagement Results

Table 3 displays engagement results. During the first campaign (August 24-30), advertisement B attracted the greatest level of engagement, including the greatest reach (10,556 people), number of impressions (12,569), reactions (221), link clicks (131), page likes (11), and opt ins (81 new community members) and the lowest opt in per cost rate (US $1.99 per opt in). This advertisement featured an image of lungs with the text “connect with others who understand what life with lung cancer is like.” Advertisements B and C were then used in the second campaign (August 31-September 6) alongside 1 new advertisement. In week 2, advertisement B again outperformed other advertisements and was subsequently implemented in week 3 (October 5-11).

During the third campaign, advertisement F, featuring the same image as advertisement B with new text “After reading some of the comments on LungCancer.net, no more self-pity, no more discouragement, as I read these comments of strength and survival and determination I feel revived” attracted the greatest number of reactions (194), comments (19), link clicks (176), and opt ins (82) at the lowest cost (US $1.10 per opt in). In the fourth campaign (November 9-15), advertisement H, with the same text as advertisement F but a simpler lung image, attracted the greatest engagement including 179 reactions, 14 page likes, and 60 opt ins at US $1.47 per opt in. In the fifth campaign (December 7-17), advertisement H was outperformed by an advertisement featuring the same image with the text, “A place to share the good, the bad, and everything in between” (advertisement I). Advertisement I attracted 114 link clicks, 22 page likes, and 50 opt ins at US $1.89 per opt in.

**Table 2:** Demographic information of those exposed to Facebook advertisements.

Age (years)	Cumulative campaign reach (N=91,835), n (%)			New members resulting from the campaign (N=863), n (%)		
	Female	Male	Unknown	Female	Male	Unknown
65+	24,005 (26.14)	5766 (6.28)	163 (0.18)	307 (35.57)	66 (7.65)	3 (0.35)
55-64	31,401 (34.29)	6572 (7.16)	181 (0.20)	257 (29.78)	63 (7.30)	3 (0.35)
45-54	13,289 (14.47)	2397 (2.61)	62 (0.07)	111 (12.86)	14 (1.62)	0 (0.00)
35-44	4427 (4.82)	975 (1.06)	20 (0.02)	23 (2.67)	1 (0.12)	0 (0.00)
25-34	138 (1.50)	404 (0.44)	12 (0.01)	7 (0.81)	2 (0.23)	0 (0.00)
18-24	591 (0.64)	115 (0.13)	15 (0.01)	6 (0.70)	0 (0.00)	0 (0.00)
Unknown	0 (0.00)	0 (0.00)	69 (0.00)	0 (0.00)	0 (0.00)	0 (0.00)

**Table 3:** Facebook advertisement engagement results.

Ad	Exposure, n		Engagement, n						Cost/opt in rate (US $)
	Reach	Impressions	Low			Medium		High (opt ins)	
			Reactions	Link clicks	Page likes	Shares	Comments		
A	7206	8972	83	81	6	16	9	34	5.10
B^a^	10,556	12,569	221	131	11	44	6	81	1.99
C	8494	10,788	219	102	10	51	8	72	2.23
B^a^	10,546	12,965	170	164	9	34	15	78	1.85
C	9326	11,944	173	113	10	37	13	55	2.64
D	6484	9235	238	121	10	35	8	61	1.86
B	6078	8091	94	116	3	35	13	48	1.89
E	4018	6293	195	126	10	22	5	60	1.51
F^a^	4778	7262	194	176	9	26	19	82	1.10
F	4711	6313	151	134	10	33	11	60	1.73
G	3468	4530	141	75	7	23	5	35	2.53
H^a^	3952	5519	179	138	14	31	9	60	1.47
H	4671	5987	189	110	14	25	13	43	2.45
I^a^	4146	5472	171	114	22	23	8	50	1.89
J	3401	5067	184	88	4	17	15	44	2.12
Total	91,835	121,007	2602	1789	149	452	157	863	2.02

^a^Signals the highest performing ad (generated the most opt ins/cost) that was subsequently used in the next ad campaign.

## Discussion

### Principal Findings

Our findings demonstrate the feasibility of utilizing Facebook advertising as a cost-efficient tool to grow online health communities. Across the 5 campaigns, 863 new members opted in to the LungCancer.net community, yielding an opt in rate (opt ins/reach) of 0.94% (863/91,835) and a cost/opt in rate of US $2.02. Although the cost-effectiveness of Facebook advertisements varies widely in recruitment literature [18-24], our cost is but slightly higher than the average cost per click of US $1.32 for health care advertisements on Facebook [25]. Although Facebook advertisements were a cost-efficient community growth tool in this study, other research provides mixed results regarding the effectiveness of Facebook advertising [18,19,26,27]. Some agree that Facebook is an efficient way to draw diverse audiences to health promotion interventions [19,26,27]. Others have found Facebook to be a useful tool to increase advertisement reach, yet the actual rate of results per reach remains low [18,26]. This may indicate that Facebook advertisements are more efficient than traditional approaches (eg, physician referral, direct mail, and email) for online community growth outside research recruitment, where strict eligibility criteria often narrow the target audience [18]. Additional research is needed to test this hypothesis and optimize strategies to grow online health communities. Although these findings do not provide for specific design recommendations to increase engagement, we found some support for promising features of advertisements that match suggestions in previous literature: use of direct quotes/testimonials [28,29]; explicit reference to social support available in the community [6]; and simple lung images that are likely to be easily interpreted as relevant [30] to those seeking lung cancer communities.

### Limitations and Future Research

Although this research provides foundational knowledge regarding the feasibility of Facebook advertisements to grow the LungCancer.net community, the findings are limited to the advertisement images and text used. Additional research is needed to systematically compare engagement with different images, texts, channels, and times of year to identify strategies associated with optimal community growth. Research is also needed to identify the impact that community growth through Facebook advertisements has on community engagement. Users who respond to a Facebook advertisement already demonstrate online engagement and may be more likely to contribute to an online health community than members recruited through other traditional strategies. Finally, given suggestions that Facebook advertising can effectively engage hardly reached populations in health education and intervention [15,18-20,27,31,32], additional research is needed to identify the sociodemographic characteristics of those engaged. Data presented here demonstrate a campaign that engaged primarily ageing female populations, representative of the current LungCancer.net site visitors (61% female and 55 years and above).

### Conclusions

This study provides a foundation for research to optimize the reach of online health communities. Facebook was a feasible, cost-effective recruitment channel for this online community, and evaluation of other advertisement designs may provide further evidence for promising engagement strategies. Online communities are vital to health promotion efforts as multiple populations seek low-cost, easily accessible health resources. Focusing on expanding the reach of such communities could have major implications for the health of future populations.
